# Overall survival according to time-of-day of combined immuno-chemotherapy for advanced gastric cancer: a propensity score-matched analysis

**DOI:** 10.3389/fimmu.2025.1653218

**Published:** 2025-12-01

**Authors:** Chong Cheng, Xiaqin Cheng, Tiejun Wang

**Affiliations:** 1 Department of Biotherapy, West China Hospital, Sichuan University, Chengdu, Sichuan, China; 2 Breast Cancer Center, Hubei Cancer Hospital, Tongji Medical College, Huazhong University of Science and Technology, Wuhan, Hubei, China; 3 National Key Clinical Specialty Discipline Construction Program, Hubei Provincial Clinical Research Center for Breast Cancer, Wuhan Clinical Research Center for Breast Cancer, Wuhan, Hubei, China

**Keywords:** immune checkpoint inhibitors, gastric cancer, chronotherapy, circadian rhythm, immunotherapy

## Abstract

**Purpose:**

The impact of time-of-day administration (ToDA) of immune checkpoint inhibitor (ICI) on patient outcomes across multiple cancer types is increasingly being elucidated. Following the results of the CheckMate-649 study, chemotherapy combined with immunotherapy has been established as the standard first-line treatment for advanced gastric cancer (GC). A thorough investigation of the relationship between ICI infusion timing and patient outcomes within this combined regimen holds considerable potential to enhance and optimize clinical treatment strategies.

**Methods:**

We conducted a retrospective analysis of patients with advanced GC from West China Hospital, Sichuan University, who received first-line chemotherapy combined with ICIs between January 2020 and September 2024. Patients who received fewer than two doses of ICIs were excluded. Follow-up continued until May 2025. The primary endpoint was overall survival (OS), defined as the time from initial ICI infusion to death from any cause. Secondary endpoints included progression-free survival (PFS), objective response rate (ORR), and adverse events (AEs). Propensity score matching (1:2 ratio, caliper width 0.1) mitigated confounding factors. The impact of ICI infusion timing (after 1630h) on OS and PFS was evaluated using Cox proportional hazards regression. ORR and AEs were assessed with either the Chi-square test or Fisher’s exact test. To further assess the robustness of our findings, sensitivity analyses were conducted using infusion proportion thresholds of 30%, 40%, and 50% at the fixed time point of 1630h, along with time-point sensitivity analyses at 30-minute intervals across the 1500h to 1700h period.

**Results:**

Among 214 patients, 50 patients received ≥20% of ICI after 1630h (Group A), while 164 patients received <20% (Group B). Before propensity score matching, patients in Group B exhibited significantly shorter OS compared to those in Group A (median 15.4 vs. 21.4 months, HR = 1.64, P = 0.014). After matching, the Group B (44 patients) continued to demonstrate significantly shorter OS than Group A (86 patients) (median 15.4 vs. 22.4 months, HR = 1.82, P = 0.013). Multivariable analysis confirmed these findings. No significant differences were found in PFS, ORR, or AEs (all P>0.050). Sensitivity analysis further validated the robustness of the results.

**Conclusion:**

Early ToDA of ICIs is associated with longer OS in patients with advanced GC receiving first-line chemotherapy combined with ICIs. Randomized clinical trials are required to validate these findings.

## 1 Introduction

Circadian rhythms constitute endogenous timing systems in organisms, characterized by 24-hour oscillations in physiological processes (1). These evolutionarily conserved mechanisms maintain organism-environment homeostasis by coordinating essential functions including sleep-wake cycles, energy metabolism, and immune responses (2–4). Chronotherapy, also known as circadian medicine, leverages endogenous circadian rhythms through administering treatments during biologically optimal time windows, thereby maximize therapeutic outcomes (5).

Previous studies have gradually elucidated the mechanisms through which circadian rhythms influence chemotherapy toxicity and the efficacy of therapeutic cytokines (6, 7). In recent years, the regulatory relationship between circadian rhythms and immune function has become increasingly well-defined. Research demonstrates that various immune cells, including monocytes, macrophages, neutrophils, and natural killer cells, possess intrinsic circadian clocks and through these regulatory mechanisms play vital roles in both innate and adaptive immune responses (8–11).Preclinical studies have demonstrated significant circadian fluctuations in the number of peripheral blood lymphocytes in healthy adults, with secretion patterns of various pro-inflammatory cytokines closely linked to cancer progression and treatment response (12, 13). CD8^+^ T cells, which are key targets of immune checkpoint inhibitors (ICIs), have recently been shown to possess intrinsic circadian rhythms, with their immune response intensity demonstrating significant diurnal variation and peaking within specific time windows (14). Collectively, these findings underscore that synchronizing immunotherapy with the body’s intrinsic circadian rhythms is critical for maximizing therapeutic efficacy.

A single-center cohort study published in 2021(MEMOIR) was the first to establish a significant correlation between the time-of-day administration (ToDA) of ICIs and patient outcomes (15). In melanoma patients, those who received ≥20% of their ICI infusions after 1630h had significantly worse survival compared to those who received <20% of infusions after this time. This finding has since been corroborated in a variety of solid tumors, including lung cancer, head and neck cancer, and renal cell carcinoma (RCC) (16–26). Real-world data from eighteen retrospective studies indicated that early ToDA of ICIs could improve progression-free survival (PFS) and overall survival (OS) by up to fourfold compared to late ToDA (27). However, research on advanced gastric cancer (GC) has been limited to nivolumab in later-line therapies (28, 29).

Given that combination chemotherapy and ICIs have been incorporated into first-line treatment guidelines for advanced GC, clarifying the impact of ICI infusion timing on survival outcomes with this specific regimen is of substantial clinical significance. The findings are expected to provide evidence-based insights for optimizing clinical dosing schedules.

## 2 Methods

### 2.1 Study design and participants

**Figure 1:**
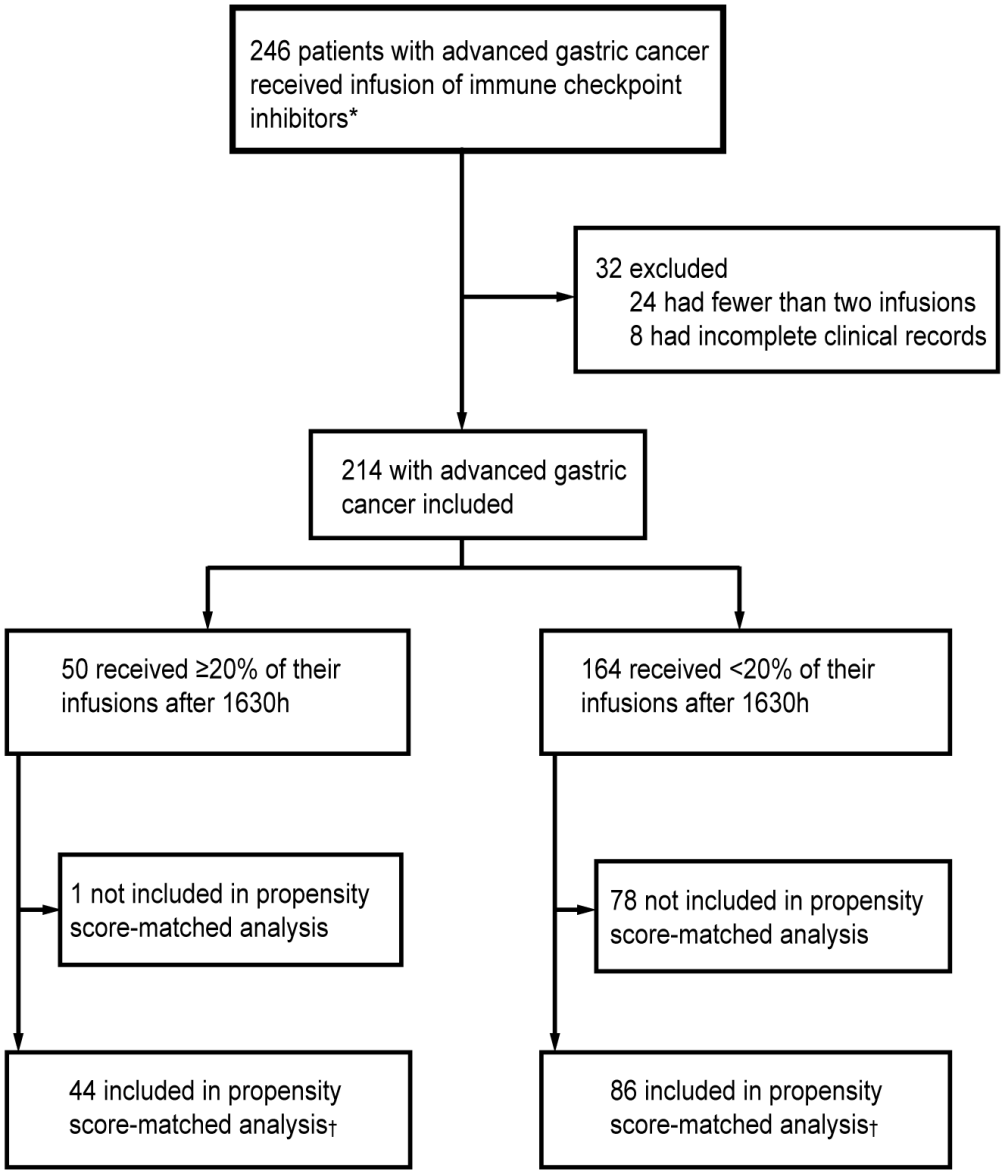
Study profile. *Started receiving infusions between January 2020 and September 2024. †Propensity score matching by concurrent targeted drugs, primary tumor location, degree of differentiation, MMR, pre-treatment CA19-9, and CA72-4.

This single-center, retrospective cohort study consecutively enrolled patients with advanced GC who received first-line chemotherapy combined with ICIs at West China Hospital, Sichuan University between January 2020 and September 2024. Patients were included if they had completed at least two cycles of ICI treatment. Follow-up data were collected until May 1, 2025. Clinical and pathological characteristics, ICI infusion timing, and survival outcomes were retrieved from the hospital’s electronic medical records. Survival data were supplemented via telephone follow-up when necessary. The key inclusion criteria were: (1) histologically confirmed gastric cancer; (2) diagnosis of locally advanced or metastatic disease at the initiation of immunotherapy; and (3) completion of at least two cycles of first-line chemotherapy combined with immunotherapy. Patients with incomplete clinical data were excluded (**Figure 1**). The study protocol was approved by the Ethics Committee of West China Hospital, Sichuan University (Approval No. 20242530).

### 2.2 Procedures

Treatment plans were determined by a multidisciplinary team. Immune checkpoint inhibitors were administered at standard regimens: Sintilimab 200mg every 3 weeks (q3w), Nivolumab 240mg q2w/360mg q3w, Pembrolizumab 200mg q3w, and Tislelizumab 200mg q3w. Doses of concomitant chemotherapy or targeted therapy were adjusted based on patient tolerance. In accordance with our institutional medication protocols, ICIs were typically administered prior to chemotherapy, unless specific circumstances dictated otherwise. Tumor response was assessed by imaging every 2–3 cycles and evaluated per RECIST version 1.1.

All treatment-related adverse events (AEs) from treatment initiation until the last follow-up were retrospectively reviewed and graded according to the Common Terminology Criteria for Adverse Events (CTCAE) version 5.0. Immune-related adverse events (irAEs) were specifically identified and classified based on the criteria established in the ORIENT-16 trial (30).

### 2.3 Outcomes

The primary endpoint was OS, defined as the time from the initiation of immunotherapy to death from any cause. Secondary endpoints included PFS, objective response rate (ORR), and AEs. PFS was defined as the time from treatment initiation to either disease progression or death from any cause, whichever occurred first. ORR was defined as the proportion of patients achieving a complete or partial response. AEs were assessed from the start of ICI treatment until the last follow-up, graded according to CTCAE version 5.0. The incidence of specific irAEs, such as thyroid dysfunction, myocarditis, and rash, was documented separately using the classification criteria from the ORIENT-16 study (30).

### 2.4 Statistical analysis

Based on previous vaccination studies and retrospective research (15, 31), patients were stratified into two groups using a cut-off point of 1630h and an infusion threshold of 20%. Group A (Early) comprised patients receiving <20% of their ICI infusions after 1630h, while Group B (Late) comprised those receiving ≥20% of infusions after this time.

To improve group comparability, we performed 1:2 propensity score matching (PSM) using logistic regression (caliper=0.1), with covariates including concurrent targeted drugs, primary tumor location, degree of differentiation, MMR, pre-treatment CA19-9, and CA72-4. Because we found a trend towards association between these factors and OS in univariate analysis (P<0.10). Balance was assessed post-matching. Survival was analyzed using Kaplan-Meier curves and Cox proportional hazards regression. Unadjusted Hazard ratios (HRs) for OS were derived from Univariate Cox proportional hazards regression in all cohorts. Multivariate Cox proportional hazards regression, adjusted for factors with P < 0.10 from univariate analysis, provided adjusted HRs for OS. PFS, ORR, and AEs were analyzed in the unmatched cohort using Chi-square or Fisher’s exact tests.

Sensitivity analyses employed alternative infusion thresholds (30%, 40%, 50%) and time points (1530h, 1600h, 1700h). All statistical analyses were performed using R software (version 4.0.3). A two-sided significance level of P < 0.05 was applied for all statistical tests.

## 3 Results

### 3.1 Patient characteristics and study flow

A total of 246 patients were initially screened. After excluding 24 patients who received fewer than two cycles of ICI and 8 with incomplete data, 214 were included in the final analysis. Based on a 20% infusion threshold after 1630h, patients were stratified into Group B (Late, n=50) and Group A (Early, n=164).

The cohort consisted of 148 patients (69%) aged <65 years and 66 (31%) aged ≥65 years, with a male predominance (144 [67%] vs. 70 [33%] female). The most commonly used ICIs were sintilimab (75%), nivolumab (13%), an additional 12% received tislelizumab, pembrolizumab, toripalimab or camrelizumab. All patients received ICIs combined with chemotherapy, primarily FOLFOX, SOX, or CAPOX regimens. The majority (194, 91%) received doublet chemotherapy, while 17 (8%) received multi-drug regimens, and 3 (1%) received monotherapy due to poor performance status. Primary tumors were located in the stomach or gastroesophageal junction, and the predominant histology was adenocarcinoma (94%), with a few cases of mixed or other types (e.g., neuroendocrine carcinoma, poorly differentiated carcinoma). 93% of patients had poorly differentiated tumors, while the remaining 7% exhibited moderately differentiated histology.

Univariate Cox regression was performed to identify prognostic factors. Following 1:2 propensity score matching, a balanced cohort of 130 patients was generated, comprising 44 in Group B and 86 in Group A. The baseline characteristics of both the unmatched and matched cohorts are presented in **Table 1**.

**Table 1:** Patient characteristics in the unmatched and propensity score-matched population.

Variable	Unmatched population		*P* value	Matched population		*P* value
	Received ≥20% of infusions after 1630 h (n=50)	Received <20% of infusions after 1630 h (n=164)		Received ≥20% of infusions after 1630 h (n=44)	Received <20% of infusions after 1630 h (n=86)	
Age			0.04^1*^			0.15^1^
<65	41 (82%)	107 (65%)		37 (84%)	61 (71%)	
≥65	9 (18%)	57 (35%)		7 (16%)	25 (29%)	
Sex			0.69^1^			0.43^1^
Male	32 (64%)	112 (68%)		28 (64%)	62 (72%)	
Female	18 (36%)	52 (32%)		16 (36%)	24 (28%)	
ECOG performance status			0.39^1^			0.33^1^
0	38 (76%)	112 (68%)		35 (80%)	60 (70%)	
≥1	12 (24%)	52 (32%)		9 (20%)	26 (30%)	
Disease stage			0.53^2^			0.72^2^
Locally advanced or recurrent	2 (4%)	12 (7%)		2 (5%)	7 (8%)	
Metastatic	48 (96%)	152 (93%)		42 (95%)	79 (92%)	
ICI			0.37^1^			0.50^2^
Nivolumab	4 (8%)	24 (15%)		3 (7%)	11 (13%)	
Sintilimab	41 (82%)	119 (73%)		36 (82%)	62 (72%)	
Others^#^	5 (10%)	21 (13%)		5 (11%)	13 (15%)	
Concurrent targeted drugs			0.76^1^			1.00^1^
Yes	13 (26%)	37 (23%)		12 (27%)	23 (27%)	
No	37 (74%)	127 (77%)		32 (73%)	63 (73%)	
Chemotherapy regimen administered			0.13^2^			0.22^2^
Single agent	0 (0%)	3 (2%)		0 (0%)	2 (2%)	
Dual therapy	49 (98%)	145 (88%)		43 (98%)	75 (87%)	
Polychemotherapy	1 (2%)	16 (10%)		1 (2%)	9 (10%)	
Primary tumor location			1.00^1^			1.00^1^
Gastric	43 (86%)	140 (85%)		37 (85%)	72 (84%)	
Gastroesophageal junction	7 (14%)	24 (15%)		7 (16%)	14 (16%)	
Histological type			0.40^2^			1.00^2^
Adenocarcinoma	46 (92%)	155 (95%)		41 (93%)	79 (92%)	
Mixed	2 (4%)	7 (4%)		2 (5%)	5 (6%)	
Others^‡^	2 (4%)	2 (1%)		1 (2%)	2 (2%)	
Degree of differentiation			0.22^2^			1.00^2^
Poorly-differentiated	44 (88%)	154 (94%)		43 (98%)	85 (99%)	
Moderately-differentiated	6 (12%)	10 (6%)		1 (2%)	1 (1%)	
Prior surgery			0.68^1^			0.77^1^
Yes	14 (28%)	39 (24%)		12 (27%)	20 (23%)	
No	36 (72%)	125 (76%)		32 (73%)	66 (77%)	
Liver metastasis			0.41^1^			0.88^1^
Yes	13 (26%)	55 (34%)		12 (27%)	26 (30%)	
No	37 (74%)	109 (66%)		32 (73%)	60 (70%)	
Peritoneal metastasis			0.02^1*^			0.16^1^
Yes	32 (64%)	72 (44%)		27 (61%)	40 (47%)	
No	18 (36%)	92 (56%)		17 (39%)	46 (53%)	
Metastasis to other sites			0.33^1^			0.15^1^
Yes	14 (28%)	33 (20%)		12 (27%)	13 (15%)	
No	36 (72%)	131 (80%)		32 (73%)	73 (85%)	
Number of metastatic organs			0.085^1^			0.082^1^
0	7 (14%)	33 (20%)		7 (16%)	19 (22%)	
1	27 (54%)	102 (62%)		23 (52%)	54 (63%)	
≥2	16 (32%)	29 (18%)		14 (32%)	13 (15%)	
MMR status			0.36^2^			1.00^2^
pMMR	47 (94%)	160 (98%)		43 (98%)	85 (99%)	
dMMR	3 (6%)	4 (2%)		1 (2%)	1 (1%)	
Her2			0.77^2^			0.73^2^
Positive	5 (10%)	13 (8%)		4 (9%)	6 (7%)	
Negative	45 (90%)	151 (92%)		40 (91%)	80 (93%)	
CEA			0.93^1^			1.00^1^
≥5ng/mL	19 (38%)	59 (36%)		16 (36%)	31 (36%)	
<5ng/mL	31 (62%)	105 (64%)		28 (64%)	55 (64%)	
CA125			0.28^1^			0.86^1^
≥24U/mL	30 (60%)	82 (50%)		25 (57%)	46 (53%)	
<24U/mL	20 (40%)	82 (50%)		19 (43%)	40 (47%)	
CA19-9			0.52^1^			1.00^1^
≥30 U/mL	14 (28%)	56 (34%)		14 (32%)	26 (30%)	
< 30U/mL	36 (72%)	108 (66%)		30 (68%)	60 (70%)	
CA72-4			1.00^1^			1.00^1^
≥6.9U/mL	31 (62%)	100 (61%)		26 (59%)	51 (59%)	
<6.9U/mL	19 (38%)	64 (39%)		18 (41%)	35 (41%)	
AFP			0.52^1^			0.63^1^
≥7ng/mL	6 (12%)	28 (17%)		5 (11%)	14 (16%)	
<7ng/mL	44 (88%)	136 (83%)		39 (89%)	72 (84%)	
NLR			0.10^1^			0.16^1^
≥3	32 (64%)	81 (49%)		27 (61%)	40 (47%)	
<3	18 (36%)	83 (51%)		17 (39%)	46 (53%)	
PLR			0.42^1^			0.15^1^
≥200	16 (32%)	65 (40%)		11 (25%)	34 (40%)	
<200	34 (68%)	99 (60%)		33 (75%)	52 (60%)	
Charlson Comorbidity Index			0.41^1^			0.46^1^
0	41 (82%)	123 (75%)		37 (84%)	66 (77%)	
≥1	9 (18%)	41 (25%)		7 (16%)	20 (23%)	
number of concomitant medication types^†^			0.07^1^			0.16^1^
0	45 (90%)	126 (77%)		39 (89%)	66 (77%)	
≥1	5 (10%)	38 (23%)		5 (11%)	20 (23%)	
Antibiotic therapy			1.00^2^			1.00^2^
Yes	1 (2%)	6 (4%)		1 (2%)	2 (2%)	
No	49 (98%)	158 (96%)		43 (98%)	84 (98%)	

ICI, Immune checkpoint inhibitor; ECOG, Eastern Cooperative Oncology Group; AFP, Alpha Fetoprotein; CA19-9, Carbohydrate Antigen 19-9; CEA, Carcinoembryonic Antigen; CA125, Carbohydrate Antigen 125; CA72-4, Carbohydrate Antigen 72-4; NLR, Neutrophil-to-Lymphocyte Ratio; PLR, Platelet-to-Lymphocyte Ratio; MMR, Mismatch Repair.

The others^#^ for ICI included tislelizumab, pembrolizumab, toripalimab and camrelizumab. The others^‡^ for histological type included neuroendocrine carcinoma and poorly differentiated carcinoma. Number of concomitant medication types^†^ included antibiotics, anticoagulants, analgesics, and hypnotics.

^1^Chi-square test; ^2^Fisher’s Exact Test. *P ≤ 0.05

### 3.2 Clinical outcome

**Figure 2:**
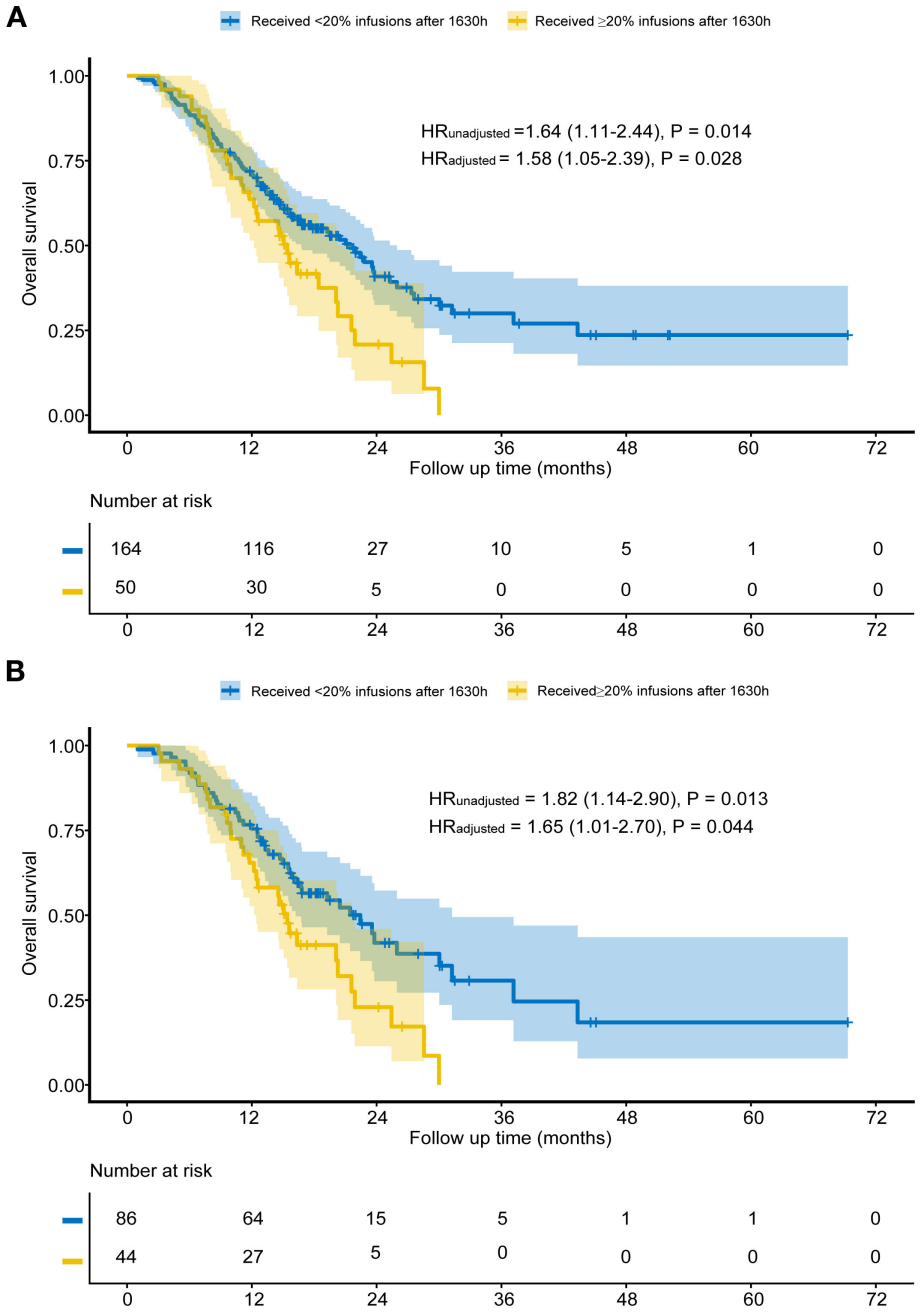
Overall survival for unmatched groups **(A)** and propensity score-matched groups **(B)**.

Before matching, the median follow-up time for patients in Group A was 14.6 months, while for those in Group B, it was 15.1 months. Kaplan-Meier analysis showed that Group B had significantly shorter OS than Group A (median 15.4 vs. 21.4 months, HR = 1.64, P = 0.014; **Figure 2A**). After adjusting for factors such as concurrent targeted drugs, primary tumor location, degree of differentiation, MMR status, pre-treatment CA19–9 and CA72-4, the adjusted HR was 1.58 (95% CI: 1.05-2.39, P = 0.028; **Figure 2A**). Multivariable cox proportional hazards regression indicated that concurrent targeted drugs was an independent prognostic factor (**Table 2**).

**Table 2:** Univariate and multivariable cox proportional hazards regression of overall survival in the unmatched population.

Variable	HR (95% CI) univariable	*P* value	HR (95% CI) multivariable	*P* value
Timing1630 (Reference= infusion<20%)				
Received ≥20% of infusions after 1630h	1.64 (1.11-2.44)	0.014	1.58 (1.05-2.39)	0.028*
Age (Reference= <65)				
≥65	0.99 (0.67-1.47)	0.978		
Sex (Reference= Male)				
Female	0.87 (0.60-1.28)	0.483		
ECOG (Reference= 0)				
≥1	1.12 (0.76-1.65)	0.554		
Disease stages (Reference=Locally recurrent/advanced)				
Metastatic	1.39 (0.61-3.16)	0.436		
ICI (Reference= Sintilimab)				
Nivolumab	0.74(0.46-1.19)	0.217		
Others^#^	0.67 (0.34-1.30)	0.236		
Concurrent targeted drugs (Reference=No)				
Yes	0.40 (0.23-0.70)	0.001	0.41 (0.24-0.72)	0.002*
Chemotherapy regimen administered (Reference= Single agent)				
Dual therapy	0.65 (0.16-2.65)	0.549		
Polychemotherapy	1.20 (0.27-5.30)	0.810		
Previous radical resection (Reference=No)				
Yes	0.96 (0.64-1.43)	0.823		
Primary tumor location (Reference= gastric)				
Gastroesophageal junction	0.62 (0.36-1.09)	0.095	0.67 (0.38-1.19)	0.173
Histological type (Reference= Adenocarcinoma)				
Mixed-type	0.66 (0.24-1.79)	0.416		
Others^‡^	0.89 (0.22-3.60)	0.867		
Degree of differentiation (Reference= Poorly-differentiate)				
Moderately-differentiated	1.96 (1.07-3.56)	0.028	1.74 (0.93-3.27)	0.083
Liver metastasis (Reference=No)				
Yes	0.80 (0.55-1.18)	0.261		
Peritoneal metastasis (Reference=No)				
Yes	1.19 (0.84-1.70)	0.331		
Metastasis to other sites (Reference=No)				
Yes	1.38 (0.93-2.06)	0.114		
Number of metastatic organs (Reference=0)				
1	0.79 (0.49-1.25)	0.311		
≥2	1.25 (0.74-2.11)	0.403		
MMR (Reference= pMMR)				
dMMR	2.23 (0.98-5.09)	0.057	1.49 (0.63-3.57)	0.366
Her2 (Reference= Negative)				
Positive	0.73 (0.35-1.49)	0.381		
CEA (Reference=<5ng/mL)				
≥5ng/mL	1.33 (0.93-1.92)	0.118		
CA125 (Reference=<24U/mL)				
≥24U/mL	1.35 (0.94-1.93)	0.100		
CA19-9(Reference=<30U/mL)				
≥30U/mL	1.46 (1.02-2.09)	0.040	1.38 (0.95-2.00)	0.095
CA72-4(Reference= <6.9U/mL)				
≥6.9U/mL	1.38 (0.95-1.99)	0.086	1.24 (0.84-1.82)	0.284
AFP (Reference= <7ng/mL)				
≥7ng/mL	1.01 (0.62-1.64)	0.979		
NLR (Reference= NLR<3)				
NLR≥3	1.12 (0.79-1.60)	0.521		
PLR (Reference= PLR<200)				
PLR≥200	0.80 (0.55-1.17)	0.245		
Charlson Comorbidity Index (Reference= 0)				
≥1	1.04 (0.68-1.59)	0.847		
number of concomitant medication types^†^ (Reference= 0)				
≥1	1.23 (0.80-1.89)	0.337		
Antibiotic therapy (Reference= No)				
Yes	0.96 (0.35-2.61)	0.935		

ICI, Immune checkpoint inhibitor; ECOG, Eastern Cooperative Oncology Group; AFP, Alpha Fetoprotein; CA19-9, Carbohydrate Antigen 19-9; CEA, Carcinoembryonic Antigen; CA125, Carbohydrate Antigen 125; CA72-4, Carbohydrate Antigen 72-4; NLR, Neutrophil-to-Lymphocyte Ratio; PLR, Platelet-to-Lymphocyte Ratio; MMR, Mismatch Repair.

The others^#^ for ICI included tislelizumab, pembrolizumab, toripalimab and camrelizumab. The others^‡^ for histological type included neuroendocrine carcinoma and poorly differentiated carcinoma. Number of concomitant medication types^†^ included antibiotics, anticoagulants, analgesics, and hypnotics.

Variables with P<0.1 were included in the multivariable cox proportional hazards regression. The adjustment factors include concurrent targeted drugs, primary tumor location, degree of differentiation, MMR, pre-treatment CA19-9, and pre-treatment CA72-4.

*P<0.05 in multivariable cox proportional hazards regression.

After matching, Group B again showed worse OS (median 15.4 vs. 22.5 months, HR = 1.82, P = 0.013; **Figure 2B**). Adjusting for the factors include concurrent targeted drugs, primary tumor location, liver metastasis, number of metastatic organs, pre-treatment CEA and CA19-9, subsequent multivariable analysis of the matched cohort confirmed the robustness of this finding (HR:1.65, 95% CI: 1.01-2.70, P = 0.044; **Figure 2B**) and revealed that concurrent targeted drugs was independent prognostic factors (**Table 3**).

**Table 3:** Univariate and multivariable cox proportional hazards regression of overall survival in the matched population.

Variable	HR (95% CI) univariable	*P* value	HR (95% CI) multivariable	*P* value
Timing1630 (Reference= infusions<20%)				
Received ≥20% infusions after 1630 h	1.82 (1.14-2.90)	0.013	1.65 (1.01-2.70)	0.044*
Concurrent targeted drugs (Reference=No)				
Yes	0.42 (0.22-0.80)	0.008	0.44 (0.21-0.91)	0.027*
Primary tumor location (Reference= gastric)				
Gastroesophageal junction	0.51 (0.26-1.03)	0.061	0.51 (0.25-1.03)	0.060
Liver metastasis (Reference= No)				
Yes	0.55 (0.33-0.93)	0.026	0.68 (0.36-1.30)	0.244
Member of metastatic organs (Reference=0)				
1	0.55 (0.32-0.97)	0.040	0.74 (0.41-1.35)	0.325
≥2	0.65 (0.33-1.25)	0.193		
CEA(Reference=<5ng/mL)				
≥5ng/mL	1.50 (0.95-2.39)	0.084	1.40 (0.80-2.46)	0.240
CA19-9 (Reference= <30U/mL)				
≥30U/mL	1.83 (1.16-2.88)	0.010	1.57 (0.88-2.80)	0.129

CA19-9, Carbohydrate Antigen 19-9; CA72-4, Carbohydrate Antigen 72-4; MMR, Mismatch Repair.

Variables with P<0.1 were included in the multivariable cox proportional hazards regression. The adjustment factors include concurrent targeted drugs, primary tumor location, liver metastasis, member of metastatic organs, pre-treatment CEA, pre-treatment CA19-9.

*P<0.05 in multivariable cox proportional hazards regression.

**Figure 3:**
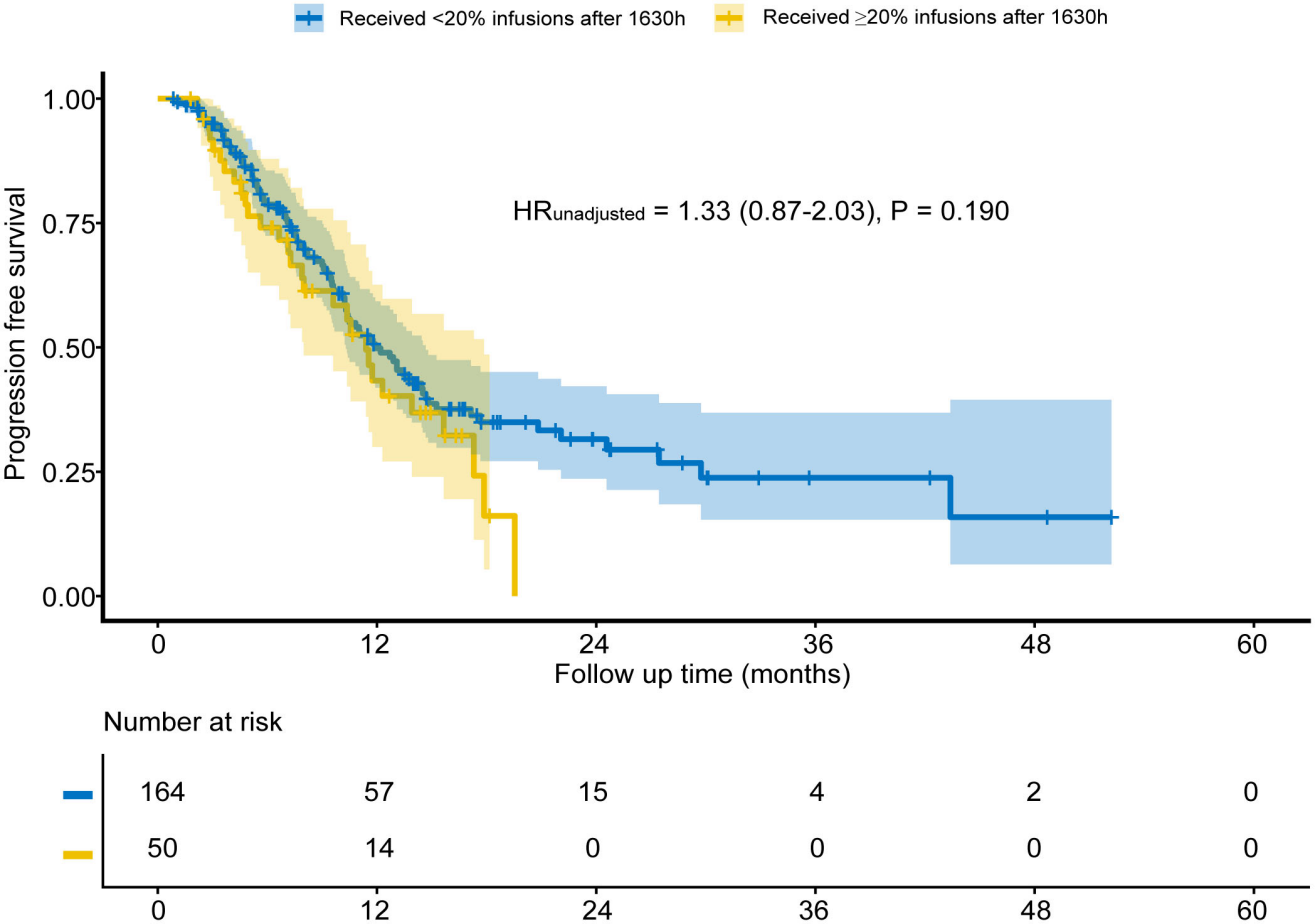
Progression-free survival for unmatched groups.

No significant between-group differences were found in PFS (HR = 1.33, P = 0.190; **Figure 3**), ORR (P = 0.653; **Table 4**), nor the incidence of any category of AEs (all P > 0.050; **Table 5**). The most frequent irAEs were thyroid dysfunction (28.5%), elevated thyroid stimulating hormone (6.5%), and myocarditis (3.7%).

**Table 4:** ORR comparison in the unmatched population.

Group	ORR, n (%)		χ2	*P* value
	Yes	No		
Received ≥20% infusions after 1630 h	18 (36%)	32 (64%)	0.202	0.653^1^
Received <20% infusions after 1630 h	67 (41%)	97 (59%)		

^1^Chi-square test; ^2^Fisher’s Exact Test.

**Table 5:** Adverse events comparison in the unmatched population.

Group	Unmatched population		χ2	*P* value
	Received ≥20% infusions after 1630 h (n=50)	Received <20% infusions after 1630 h (n=164)		
AE	45 (90%)	149 (91%)	0.000	1.000^1^
Grade 3/4 AE	14 (28%)	49 (30%)	0.006	0.938^1^
irAE	22 (44%)	61 (37%)	0.488	0.485^1^
Grade 3/4 irAE	0 (0)	2 (1%)	/	1.000^2^
AE leading to discontinuation of medication	0 (0)	6 (4%)	/	0.340^2^

AE, Adverse event; irAE, Immune-related adverse event.

^1^Chi-square test; ^2^Fisher’s Exact Test.

### 3.3 Sensitivity analysis

#### 3.3.1 Sensitivity analysis of different proportion thresholds (1630h)

To validate the robustness of our findings, we further evaluated the impact of different infusion proportion thresholds (30%, 40%, and 50%) at 1630h on clinical outcomes.

In the pre-matched cohort, all three thresholds were significantly associated with OS:30% (HR:2.15, 95% CI: 1.39–3.33, P < 0.001), 40% (HR: 2.01, 95% CI: 1.24–3.26, P = 0.004), and 50% thresholds (HR: 2.37, 95% CI: 1.43–3.92, P < 0.001) (**Supplementary Table S1**). These associations remained significant via multivariate adjustment, with adjusted HRs of 2.08 (95% CI: 1.33–3.25, P = 0.001), 1.93 (95% CI: 1.19–3.15, P = 0.008), and 2.30 (95% CI: 1.38–3.82, P = 0.001), respectively (**Supplementary Table S1**).

In the propensity score-matched cohort, significant associations with OS were similarly observed at the 30% (HR: 1.99, 95% CI: 1.17–3.40, P = 0.011), 40% (HR: 1.94, 95% CI: 1.05–3.59, P = 0.035), and 50% thresholds (HR: 2.16, 95% CI: 1.15–4.07, P = 0.017) (**Supplementary Table S2**). After multivariable adjustment, the corresponding HRs were 2.17 (95% CI: 1.22–3.85, P = 0.009), 2.26 (95% CI: 1.18–4.31, P = 0.014), and 2.61 (95% CI: 1.34–5.08, P = 0.005), respectively (**Supplementary Table S2**).

In the unmatched cohort, significant correlations with PFS were observed at the 30% (HR: 1.66, 95% CI: 1.01–2.73, P = 0.045), 40% (HR: 1.88, 95% CI: 1.11–3.20, P = 0.020), and 50% thresholds (HR: 2.48, 95% CI: 1.41–4.36, P = 0.002) (**Supplementary Table S3**). These remained significant after multivariable adjustment (all P < 0.05; **Supplementary Table S3**). However, no significant differences in ORR or irAEs were observed at any threshold (all P > 0.05; **Supplementary Tables S4**, **S5**).

#### 3.3.2 Sensitivity analysis of different time cut-off points

We further analyzed the impact of ICI infusion timing using 1530h, 1600h, and 1700h as alternative cut-off points.

In the unmatched sample, OS showed no significant association with infusion timing at 1530h. However, significant associations were observed at 1600h (HR;1.49, 95% CI: 1.03-2.15, P = 0.035) and 1700h (HR: 1.99, 95% CI: 1.24-3.20, P = 0.005) (**Supplementary Table S6**). OS was significantly associated infusion timing. These findings were confirmed in multivariable Cox regression analyses for the unmatched cohort, with adjusted HRs of 1.63 (95% CI: 1.11-2.37, P = 0.012) at 1600h and 1.79 (95% CI: 1.06-3.01, P = 0.029) at 1700h (**Supplementary Table S6**).

In the propensity score-matched cohort, infusion timing was significantly associated with OS at 1530h (HR: 1.74, 95% CI: 1.17-2.59, P = 0.007), 1600h (HR: 1.80, 95% CI: 1.16-2.79 P = 0.009), and 1700h (HR: 1.91, 95% CI: 1.01-3.62, P = 0.047) (**Supplementary Table S7**). Subsequent multivariable analysis of the matched cohort yielded adjusted hazard ratios of 1.72 (95% CI: 1.13-2.61; P = 0.012) for 1530h, 1.77(95% CI: 1.09-2.86, P = 0.021) for 1600h, and 1.88 (95% CI: 0.99-3.57, P = 0.053) for 1700h (**Supplementary Table S7**).

In the unmatched cohort, only the 1700h cut-off was significantly associated with PFS (HR: 1.82, 95% CI: 1.08-3.06, P = 0.024), and this persisted after multivariable adjustment (HR: 1.81, 95% CI: 1.05-3.09, P = 0.031; **Supplementary Table S8**). No significant differences were observed for 1530h and 1600h. No differences in ORR or irAEs were observed at any cut-off point (all P > 0.050; **Supplementary Tables S9**, **S10**).

## 4 Discussion

This retrospective study demonstrates a significant association between the timing of ICI administration and survival outcomes in patients with advanced GC receiving first-line immunotherapy combined with chemotherapy. The primary analysis revealed that in the unmatched cohort, patients who received ≥20% of their ICI infusions after 1630h had significantly shorter overall survival. This finding remained consistent after controlling for potential confounding factors through propensity score matching.

Sensitivity analyses further confirmed the robustness of our findings. When using 1630h as the reference time point with different thresholds (30%, 40%, and 50%), all thresholds demonstrated significant associations with both OS and PFS in the pre-matched cohort. These associations remained statistically significant in the matched cohort. Furthermore, analyses using alternative time cut-offs (1530h, 1600h, and 1700h) revealed significant associations with OS in the matched cohort. It should be noted that for the 1700h cut-off, the substantial sample size disparity in the pre-matched cohort and the subsequent reduction in effective sample size after 1:2 propensity score matching warrant cautious interpretation. The generalizability of these particular findings requires validation in prospective studies.

The biological plausibility of our findings is supported by a growing body of evidence on circadian immunology. Previous studies have demonstrated that the timing of vaccination could influence the magnitude of antibody responses; for instance, morning administration of influenza or COVID-19 vaccines has been associated with a more robust antiviral antibody response (32, 33). The circadian clock regulates innate immunity by controlling the rhythmic production and transport of cytokines and chemokines, as well as the maturation and tissue infiltration of immune cells. Within 24 hours following antibody infusion, tumor-infiltrating CD8^+^ T cells exhibit significant time-dependent phenotypic alterations, indicating their participation in the primary circadian response. It is noteworthy that the antitumor effect was significantly higher in the morning compared to the afternoon (34). Studies in immunodeficient mice have shown that dendritic cells (DCs) rhythmically migrate to tumor-draining lymph nodes, thereby orchestrating the circadian rhythm of tumor antigen-specific CD8^+^ T cell responses (35). This mechanism indicates that synchronizing immunotherapy with the peak functional phase of DCs could significantly enhance its antitumor efficacy. Conversely, disruption of the epithelial cell circadian clock alters cytokine secretion, exacerbates inflammatory responses, promotes neutrophil infiltration, and can ultimately lead to the expansion of myeloid-derived suppressor cells (MDSCs) (36). Importantly, the therapeutic efficacy of PD-L1 inhibitors appears to be optimal when administered in alignment with the circadian fluctuations of MDSCs (36). Consequently, strategically timing immune system activation based on circadian principles may not only maximize the intensity of immune responses but also optimize antitumor treatment outcomes.

Two previous retrospective studies investigating nivolumab as a later-line treatment for GC both showed superior survival outcomes for patients receiving morning infusions (28, 29). Following the publication of the CheckMate649 trial, the era of first-line chemotherapy combined with immunotherapy became the standard of care for advanced GC (37). This regimen has improved mOS to approximately 15 months, compared to about 10 months with chemotherapy alone. Subsequent trials, including ORIENT-16, RATIONALE-305, and GEMSTONE-303, have further established the efficacy of Sintilimab, Tislelizumab, and Sugemalimab in first-line setting (30, 38, 39). Given the prevalent use of sintilimab in our clinical practice and study cohort, we performed a subgroup analysis focusing on patients receiving sintilimab plus chemotherapy. In the unmatched sintilimab subgroup, a significant association between infusion timing and OS was observed, which persisted after multivariable adjustment. However, in the propensity score-matched sintilimab subgroup, univariate analysis indicated only a borderline significant association (HR:1.53, 95% CI: 0.94-2.49, P = 0.088), which lost statistical significance after multivariable adjustment (HR:1.50, 95% CI: 0.92-2.45, P = 0.101).This discrepancy may be attributed to several factors: First, while propensity score matching balanced known prognostic variables, there may be unmeasured confounding variables, unmeasured confounders including tumor microenvironment heterogeneity, genomic profiles, or lifestyle differences may persist. The influence of such residual confounding can be magnified in subgroup analyses, potentially obscuring a true treatment effect. Second, the reduced sample size post-matching limited statistical power, increasing the risk of a Type II error. Notably, the hazard ratios from both univariate and multivariate analyses consistently ranged between 1.50 and 1.53, suggesting a stable, albeit not statistically significant, effect size. Although the P-values slightly exceeded the conventional threshold, this consistent trend may still hold clinical relevance, particularly in light of established circadian regulation of immune function.

Previous studies have reported circadian rhythm and gender differences in immune therapy responses, with female patients showing better prognoses than males (26, 40, 41). For instance, studies of hepatitis A and influenza vaccines have shown that female individuals often mount stronger peak antibody responses (42). This heightened response may be partly attributed to sex-specific resilience to immune dysfunction induced by sleep disturbances and psychological stress (43). Additionally, women tend to display more pronounced circadian rhythmicity, characterized by higher plasma levels of melatonin and cortisol (44, 45). In contrast, a separate systematic review and meta-analysis demonstrated that while ICIs improve overall survival in patients with advanced cancers like melanoma and non-small cell lung cancer, the survival benefit was more pronounced in male patients (46). In our study, we found no significant association between patient sex and survival outcomes. Further research is warranted to clarify the potential role of sex as a biological variable and its interaction with circadian timing in shaping immunotherapy efficacy.

Our study has several limitations. First, although we observed a significant benefit in OS, no between-group differences were observed in PFS or ORR. This discrepancy may be explained by several factors. Patients in the control group might have received subsequent immunotherapy or other effective treatments after disease progression. Such crossover could mitigate between-group differences in PFS while concurrently improving long-term survival in the control group. Additionally, pseudoprogression or delayed clinical responses may not be accurately captured by conventional RECIST criteria, thereby influencing PFS and ORR assessments. The relatively limited sample size, coupled with closely matched median PFS between groups, may also have reduced the statistical power to detect a significant difference in PFS. Second, thresholds for defining early and late ICI infusion vary across previous studies, with cutoff times ranging from 1137h to 1630h and infusion proportion thresholds from 20% to 75% (27, 33). The 1630h cut-off adopted in our primary analysis is the most commonly applied, potentially due to its alignment with findings from earlier vaccination studies. To better identify optimal daily infusion timing, Catozzi et al. proposed a circadian mortality risk model; however, this model was derived from a heterogeneous cohort encompassing multiple cancer types and treatment lines (26). While our sensitivity analyses investigated various infusion cut-offs, they were insufficient to pinpoint a single optimal administration time. This highlights the need for future research specifically designed to identify the most therapeutic time window. Previous studies often determined assessment timing based on initial imaging or overall patient condition and typically excluded patients receiving fewer than four ICI doses (47, 48). In our study, considering standard efficacy assessment practices in China and the generally good performance status (ECOG score ≤1) of enrolled patients, we included all patients who received at least two infusions. The persistence of therapeutic antibody effects over weeks suggests that circadian regulation during the initial ICI cycles may be clinically more impactful than in later cycles. Thus, our study design is particularly relevant for evaluating the effect of infusion timing during the early phase of immunotherapy. Including patients with fewer infusion cycles also improves the real-world applicability of our findings, as early treatment discontinuation due to toxicity or progression is common in clinical practice. We nevertheless acknowledge that this approach introduces limitations, including relatively limited treatment exposure and potential heterogeneity in immune responses, which may affect the robustness of the results.

Adjusting the administration timing of ICIs to the biologically favorable morning window may not only enhance antitumor efficacy but also improve healthcare resource allocation and patient throughput. In accordance with our institutional treatment pathway, an “ICI-first” sequence is generally adopted, wherein immunotherapy is administered prior to chemotherapy. This approach aims to fully activate the immune system before it is subjected to chemotherapy-induced suppression, thereby potentially improving the body’s tolerance to and recovery from the immunotoxic effects of subsequent chemotherapy. However, uniformly concentrating all ICI infusions in the morning hours presents two major challenges. First, it fails to account for individual differences in circadian rhythm phenotypes, which may diminish the potential benefits of chronotherapy for some patients. Second, such highly consolidated scheduling can lead to overutilization of infusion center capacity, nursing staff, and pharmacy resources during the morning, while resulting in underutilization in the afternoon, ultimately undermining sustained gains in operational efficiency. Therefore, future implementation of chronotherapy-based ICI administration requires careful consideration of both individual chronobiological profiles and institutional resource management to optimize therapeutic outcomes while maintaining healthcare system sustainability.

## 5 Conclusions

In conclusion, our study demonstrates a significant link between earlier ICI infusion timing and improved survival in advanced gastric cancer patients treated with first-line chemoimmunotherapy. Confirming this association in prospective trials and elucidating the specific circadian mechanisms involved are critical next steps.

## Data Availability

The raw data supporting the conclusions of this article will be made available by the authors, without undue reservation.
