# Supplementary material for: Overall survival according to time-of-day of combined immuno-chemotherapy for advanced gastric cancer: a propensity score-matched analysis
Source: Front Immunol. 2025 Dec 1;16:1653218. doi: 10.3389/fimmu.2025.1653218 (PMC12702900; doi:10.3389/fimmu.2025.1653218)
Supplement: Supplementary file 1 [file Table1.docx]

**Supplementary tables**

**TableS1** **Sensitivity analysis of overall survival with different proportion thresholds after 1630h (Unmatched population)**

| **Proportion thresholds** | **HR (95% CI)**  **univariable** | ***P* value** | **HR (95% CI)**  **multivariable** | ***P* value** |
| --- | --- | --- | --- | --- |
| ≥20% infusions after 1630 vs <20% infusions after 1630h | 1.64 (1.11-2.44) | 0.014 | 1.58 (1.05-2.39) | 0.028* |
| ≥30% infusions after 1630 vs <30% infusions after 1630h | 2.15 (1.39-3.33) | <0.001 | 2.08 (1.33-3.25) | 0.001* |
| ≥40% infusions after 1630 vs <40% infusions after 1630h | 2.01 (1.24-3.26) | 0.004 | 1.93 (1.19-3.15) | 0.008* |
| ≥50% infusions after 1630 vs <50% infusions after 1630h | 2.37 (1.43-3.92) | <0.001 | 2.30 (1.38-3.82) | 0.001* |

*P<0.05 in multivariable cox proportional hazards regression.

**TableS2** **Sensitivity analysis of overall survival with different proportion thresholds after 1630h**

**(matched population)**

| **Proportion thresholds** | **HR (95% CI)**  **univariable** | ***P* value** | **HR (95% CI)**  **multivariable** | ***P* value** |
| --- | --- | --- | --- | --- |
| ≥20% infusions after 1630 vs <20% infusions after 1630h | 1.82 (1.14-2.90) | 0.013 | 1.65 (1.01-2.70) | 0.044* |
| ≥30% infusions after 1630 vs <30% infusions after 1630h | 1.99 (1.17-3.40) | 0.011 | 2.17 (1.22-3.85) | 0.009* |
| ≥40% infusions after 1630 vs <40% infusions after 1630h | 1.94 (1.05-3.59) | 0.035 | 2.26 (1.18-4.31) | 0.014* |
| ≥50% infusions after 1630 vs <50% infusions after 1630h | 2.16 (1.15-4.07) | 0.017 | 2.61 (1.34-5.08) | 0.005* |

*P<0.05 in multivariable cox proportional hazards regression.

**TableS3 Sensitivity analysis of progression-free survival with different proportion thresholds after 1630h**

**(unmatched population)**

| **Proportion thresholds** | **HR (95% CI)**  **univariable** | ***P* value** | **HR (95% CI)**  **multivariable** | ***P* value** |
| --- | --- | --- | --- | --- |
| ≥20% infusions after 1630 vs <20% infusions after 1630h | 1.33 (0.87-2.03) | 0.190 | **/** | **/** |
| ≥30% infusions after 1630 vs <30% infusions after 1630h | 1.66 (1.01-2.73)/ | 0.045 | 1.72 (1.03-2.88) | 0.038* |
| ≥40% infusions after 1630 vs <40% infusions after 1630h | 1.88 (1.11-3.20) | 0.020 | 1.93 (1.12-3.34) | 0.019* |
| ≥50% infusions after 1630 vs <50% infusions after 1630h | 2.48 (1.41-4.36) | 0.002 | 2.79 (1.58-4.95) | <0.001* |

*P<0.05 in multivariable cox proportional hazards regression.

**TableS4 Sensitivity analysis of ORR with** **different** **proportion thresholds after 1630h**

**(unmatched population)**

| **Proportion thresholds** | **≥X% infusions** | **<X% infusions** | ****$\boldsymbol{\chi}^{\boldsymbol{2}}$ | ***P* value** |
| --- | --- | --- | --- | --- |
| 20% | 18(36%) | 67(41%) | 0.202 | 0.653^1^ |
| 30% | 10 (31%) | 75 (41%) | 0.750 | 0.387^1^ |
| 40% | 7 (28%) | 78 (41%) | 1.117 | 0.291^1^ |
| 50% | 6 (30%) | 79 (41%) | 0.480 | 0.488^1^ |

1. Chi-square test; 2. Fisher's Exact Test.

**TableS5 Sensitivity analysis of irAE with different proportion thresholds after 1630h**

**(unmatched population)**

| **Proportion thresholds** | **≥X% infusions** | **<X% infusions** | ****$\boldsymbol{\chi}^{\boldsymbol{2}}$ | ***P* value** |
| --- | --- | --- | --- | --- |
| 20% | 22(44%) | 61(37%) | 0.488 | 0.485^1^ |
| 30% | 13(41%) | 70(38%) | 0.001 | 0.972^1^ |
| 40% | 10(40%) | 73(39%) | 0.000 | 1.000^1^ |
| 50% | 9(45%) | 74(38%) | 0.128 | 0.720^1^ |

1. Chi-square test; 2. Fisher's Exact Test.

**TableS6 Sensitivity analysis of overall survival in the unmatched population**

| **Cut-off points** | **HR (95% CI)**  **univariable** | ***P* value** | **HR (95% CI)**  **multivariable** | ***P* value** |
| --- | --- | --- | --- | --- |
| ≥20% infusions after 1530 vs <20% infusions after 1530h | 1.30 (0.91-1.86） | 0.156 | / | / |
| ≥20% infusions after 1600 vs <20% infusions after 1600h | 1.49 (1.03-2.15） | 0.035 | 1.63 (1.11-2.37） | 0.012* |
| ≥20% infusions after 1630 vs <20% infusions after 1630h | 1.64 (1.11-2.44） | 0.014 | 1.58 (1.05-2.39） | 0.028* |
| ≥20% infusions after 1700 vs <20% infusions after 1700h | 1.99 (1.24-3.20） | 0.005 | 1.79 (1.06-3.01） | 0.029* |

*P<0.05 in multivariable cox proportional hazards regression.

**TableS7 Sensitivity analysis of overall survival in the matched population**

| **Cut-off points** | **HR (95% CI)**  **univariable** | ***P* value** | **HR (95% CI)**  **multivariable** | ***P* value** |
| --- | --- | --- | --- | --- |
| ≥20% infusions after 1530 vs <20% infusions after 1530h | 1.74 (1.17-2.59） | 0.007 | 1.72 (1.13-2.61） | 0.012* |
| ≥20% infusions after 1600 vs <20% infusions after 1600h | 1.80 (1.16-2.79) | 0.009 | 1.77 (1.09-2.86) | 0.021* |
| ≥20% infusions after 1630 vs <20% infusions after 1630h | 1.82 (1.14-2.90） | 0.013 | 1.65 (1.01-2.70） | 0.044* |
| ≥20% infusions after 1700 vs <20% infusions after 1700h | 1.91 (1.01-3.62） | 0.047 | 1.88 (0.99-3.57） | 0.053* |

*P<0.05 in multivariable cox proportional hazards regression.

**TableS8 Sensitivity analysis of** **progression-free survival in the unmatched population**

| **Cut-off points** | **HR (95% CI) univariable** | ***P* value** | **HR (95% CI) multivariable** | ***P* value** |
| --- | --- | --- | --- | --- |
| ≥20% infusions after 1530 vs <20% infusions after 1530h | 0.91 (0.63-1.31) | 0.610 | / | / |
| ≥20% infusions after 1600 vs <20% infusions after 1600h | 1.11 (0.76-1.61) | 0.601 | / | / |
| ≥20% infusions after 1630 vs <20% infusions after 1630h | 1.33 (0.87-2.03) | 0.190 | / | / |
| ≥20% infusions after 1700 vs <20% infusions after 1700h | 1.82 (1.08-3.06） | 0.024 | 1.81 (1.05-3.09) | 0.031* |

*P<0.05 in multivariable cox proportional hazards regression.

**TableS9 Sensitivity analysis of ORR in the unmatched population**

| **Cut-off points** | **≥20% infusions** | **<20% infusions** | ****$\boldsymbol{\chi}^{\boldsymbol{2}}$ | ***P* value** |
| --- | --- | --- | --- | --- |
| after 1530h | 47(47%) | 38(34%) | 3.191 | 0.074^1^ |
| after 1600h | 39(48%) | 46(35%) | 3.321 | 0.068^1^ |
| after 1630h | 18(36%) | 67(41%) | 0.202 | 0.653^1^ |
| after 1700h | 10(36%) | 75(40%) | 0.066 | 0.797^1^ |

1.Chi-square test; 2. Fisher's Exact Test.

**TableS10 Sensitivity analysis of irAE in the unmatched population**

| **Cut-off points** | **≥20% infusions** | **<20% infusions** | $\boldsymbol{\chi}^{\boldsymbol{2}}$ | ***P* value** |
| --- | --- | --- | --- | --- |
| after 1530h | 44(44%） | 39(35%) | 1.479 | 0.224^1^ |
| after 1600h | 35(43%) | 48(36%) | 0.796 | 0.372^1^ |
| after 1630h | 22(44%) | 61(37%) | 0.488 | 0.485^1^ |
| after 1700h | 12(43%) | 71(38%) | 0.071 | 0.790^1^ |

1.Chi-square test; 2. Fisher's Exact Test.
